# Development and uptake of an online systematic review platform: the early years of the CAMARADES Systematic Review Facility (SyRF)

**DOI:** 10.1136/bmjos-2020-100103

**Published:** 2021-03-30

**Authors:** Zsanett Bahor, Jing Liao, Gillian Currie, Can Ayder, Malcolm Macleod, Sarah K McCann, Alexandra Bannach-Brown, Kimberley Wever, Nadia Soliman, Qianying Wang, Lee Doran-Constant, Laurie Young, Emily S Sena, Chris Sena

**Affiliations:** 1 Centre for Clinical Brain Sciences, The University of Edinburgh, Edinburgh, Scotland, UK; 2 Charité - Universitätsmedizin Berlin, corporate member of Freie Universität Berlin, Humboldt-Universität zu Berlin, and Berlin Institute of Health, Berlin, Germany; 3 QUEST – Center for Transforming Biomedical Research, Berlin Institute of Health (BIH), Berlin, Germany; 4 Institute for Evidence-Based Practice, Bond University, Robina, Queensland, Australia; 5 Systematic Review Centre for Laboratory animal Experimentation (SYRCLE), Department for Health Evidence, Nijmegen Institute for Health Sciences, Radboud University Medical Center, Nijmegen, The Netherlands; 6 Pain Research, Imperial College London, London, UK; 7 Independent Researcher, Manchester, UK; 8 Frozen Event Ltd, London, UK

**Keywords:** systematic review, preclinical research, automation

## Abstract

Preclinical research is a vital step in the drug discovery pipeline and more generally in helping to better understand human disease aetiology and its management. Systematic reviews (SRs) can be powerful in summarising and appraising this evidence concerning a specific research question, to highlight areas of improvements, areas for further research and areas where evidence may be sufficient to take forward to other research domains, for instance clinical trial. Guidance and tools for preclinical research synthesis remain limited despite their clear utility. We aimed to create an online end-to-end platform primarily for conducting SRs of preclinical studies, that was flexible enough to support a wide variety of experimental designs, was adaptable to different research questions, would allow users to adopt emerging automated tools and support them during their review process using best practice. In this article, we introduce the Systematic Review Facility (https://syrf.org.uk), which was launched in 2016 and designed to support primarily preclinical SRs from small independent projects to large, crowdsourced projects. We discuss the architecture of the app and its features, including the opportunity to collaborate easily, to efficiently manage projects, to screen and annotate studies for important features (metadata), to extract outcome data into a secure database, and tailor these steps to each project. We introduce how we are working to leverage the use of automation tools and allow the integration of these services to accelerate and automate steps in the systematic review workflow.

**Strengths and limitations of this study**

- Systematic Review Facility (SyRF) is flexible and scalable to accommodate different project types.
- SyRF allows collaborative teams to screen studies independently, blinded to other reviewers, and regardless of geographical location.
- Several SyRF projects have successfully used automated techniques such as machine learning and text mining as part of their systematic review process.
- We support our users in best practise through educational materials and more bespoke one-on-one methodological guidance for preclinical reviews.
- SyRF has been released with minimum features and continuous development will be required to add functionality guided by user demand and feedback.

## Introduction

The last decade has seen a substantial increase in the conduct and reporting of systematic reviews of preclinical data; PubMed identifies 512 non-human systematic reviews published up to 2013,^1^ rising to over 2500 up to 2019 (search September 2020). Since 2005, the Collaborative Approach to Meta-Analysis and Review of Animal Data from Experimental Studies (CAMARADES; www.camarades.info) group have provided training and mentoring to those wishing to conduct systematic reviews and meta-analyses of data from preclinical studies. In response to challenges encountered while conducting reviews, in particular, in ensuring efficient, robust and reproducible data and process management, we moved away from a local database (most recently enabled using Microsoft Access, and accessible through a Remote Desktop session) to a cloud-based approach. Here, we outline the basic structure of the CAMARADES Systematic Review Facility (SyRF), along with a description of current features, features in development and usage statistics. Our purposes here are, first, to describe the features of SyRF such that those wishing to conduct a systematic review of preclinical data are aware of the functions available so that they can use them to their fullest potential; and so that those developing automation tools in this area can understand the basics of the automation approach taken for each task. We do not aim to make the case for why systematic reviews of preclinical data are important (this will be described in another paper in this series); to provide a detailed technical description of the coding involved (which will be released on our GitHub repository) or to present a user guide (which we provide at https://assets.syrf.org.uk/guides/SyRF_User_Guide.pdf).

**Figure 1:**
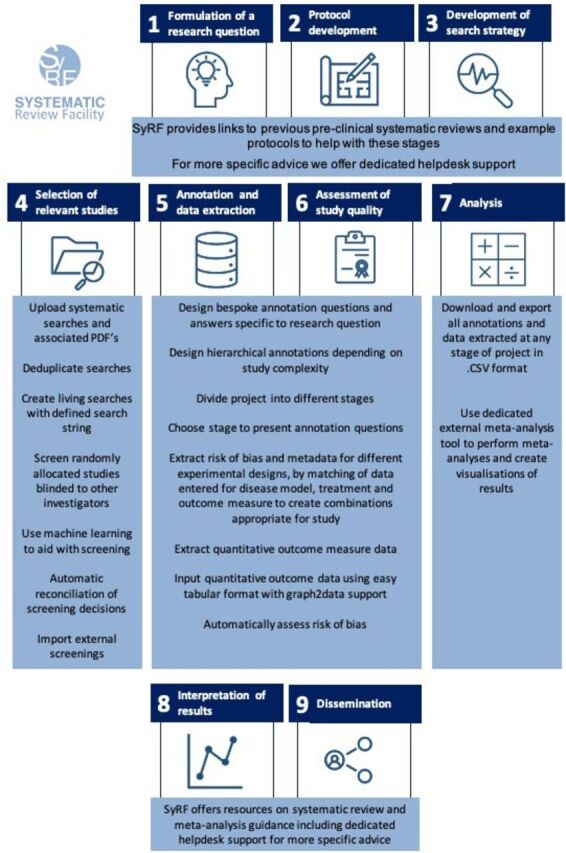
Systematic review workflow diagram. SyRF, Systematic Review Facility.

A typical systematic review workflow includes nine steps: (1) formulation of a research question, (2) protocol development, (3) development of a search strategy, (4) selection of relevant studies, (5) metadata annotation and quantitative outcome data extraction, (6) assessment of study quality, (7) analysis, (8) interpretation of results and (9) dissemination. As shown in figure 1, SyRF provides online methodological guidance for each of these steps as well as a dedicated support team.

We developed SyRF to address several challenges we encountered when performing systematic reviews ourselves. First, we previously screened citations for inclusion by independent reviewers working through a list of records in a bibliographic platform such as Reference Manager or Endnote. Their results then had to be combined, and discrepancies identified and passed to a third reviewer. Having more than two individuals acting as primary reviewers was complicated, and it was not possible to easily associate screening decisions with reviewers or monitor progress and screening activity.

Following screening, each included publication had to be manually entered to Microsoft Excel or later a Microsoft Access database (giving opportunities for transcribing errors), and annotations of study metadata and quantitative outcome data reported in each publication extracted to fixed fields. The CAMARADES Microsoft Access database was hosted on an internal University of Edinburgh server, and the involvement of external collaborators was limited by the administrative requirement that they be given access to our systems. Included publications had a project level but not a system-wide unique identifier, the individual responsible for data items entered was not known, and data could be overwritten or deleted in error, with no audit trail to identify that this had happened. Preclinical research data are often presented graphically, and so those doing data extraction would use virtual or physical rulers to measure distances, convert these to values, and then enter them to the system.

With no workflow control, deploying effort among collaborative teams was exceedingly challenging, especially when some team members were accessing the project from the other side of the world, through the remote desktop and with suboptimal internet bandwidth.

Human errors in citation screening, study metadata and quantitative data extraction are widely recognised, and to mitigate these errors, it is usual practice to require that two people independently conduct every task, with differences resolved by a third person. Independent metadata annotation of studies and extraction of outcome data from these studies required duplicate projects to be created and then merged. This process of reconciliation was complex and could not be documented efficiently.

To evaluate studies in aggregate (including meta-analysis), data had to be exported to statistical software, again giving opportunities for error. Initially, meta-analysis was conducted in Microsoft Excel, which at least had the advantage that users had to understand the maths behind their analysis, but again this did not support a reproducible or curated workflow.

The user experience was disjointed and unsatisfactory. Reviewer training was ad hoc, with no support for training projects. It was not possible to measure the progress or performance (eg, agreement with other reviewers), and on the very rare occasions where the performance of a reviewer was found to fall below expected standards, it was not possible to identify which of the recorded decisions they had made, meaning that all decisions had to be checked.

Finally, while not of practical concern in the early years, the disjointed approach was not conducive to the implementation of processes to support automation of for instance searching, deduplication, citation screening, risk of bias annotation or meta-analysis. It made the work of systematic reviews very burdensome and limited the appropriate use of the methodology to a few small groups with the experience, capacity and confidence to proceed.

In light of the challenges faced by our group and many of our collaborators, SyRF grew to meet the need for a bespoke, simple and free to use software to support our performance of systematic reviews of preclinical studies. Since its first deployment in 2016, SyRF has undergone substantial improvement (current v2.4.8).

## Architecture of SYRF

SyRF was conceived as an open-access web interface supporting end-to-end systematic reviews of preclinical data. While developed for reviews of efficacy studies using animal models of human disease, it is sufficiently flexible to be used for any systematic review.

**Figure 2:**
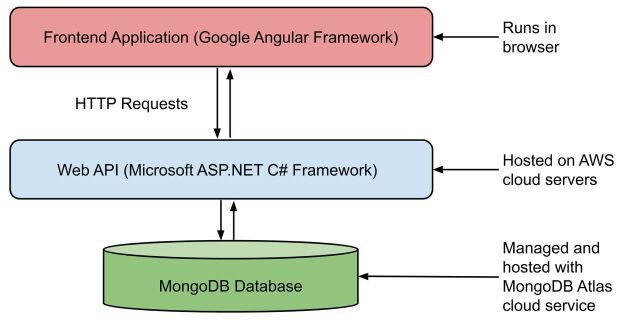
Systematic Review Facility architecture diagram.

SyRF offers a secure platform to extract and store data for systematic reviews. As demonstrated in figure 2, SyRF infrastructure includes application data, a web API and a frontend application. This infrastructure is horizontally scalable to meet an increase in demand of systematic reviews.

The user interface for SyRF is provided through a client-side single-page application created using Google’s Angular Framework. This allows the user interface to be improved and updated independently from the rest of the application. This is particularly relevant for the API which requires increased stability as it acts as an extensibility point.

The web API was developed using Microsoft’s ASP.NET Core technology and is hosted in the cloud on an AWS web server. The SyRF API consists of multiple endpoints that control all business logics of the SyRF application. These endpoints are designed to receive HTTP requests from the Angular web app. The endpoints also provide a point of extensibility by allowing external applications to communicate with SyRF using HTTP requests. Security and authorisation are maintained by requiring all requests to contain an authentication token in the header.

Application data are securely held, maintained and backed up every 6 hours using an encrypted cloud solution managed by MongoDB cloud service. MongoDB is suited to storing huge collections of data while maintaining speed and availability through efficient use of indexes and clusters. Data are only accessed and mutated via the web API, and the various clients (frontend applications, plugins and extensions) do not have direct access to the MongoDB database cluster. This is by design to maintain data integrity and security.

## Systematic review workflow

We help individuals formulate their research question. We provide example protocols on our site to help users set out in advance how they will conduct their review including how data will be analysed. We strongly recommend publishing or registering a protocol (eg, at PROSPERO, the international database for SR protocols; https://www.crd.york.ac.uk/prospero/) before initiating a systematic review as it helps others know that work is ongoing (and therefore avoids duplications), allows others to make suggestions for improvement, and gives confidence to readers of systematic reviews that your study design and analysis plan preceded data collection. We created the platform intending to promote open science, transparency and high-quality research.

We highly encourage completing the first three steps before formally initiating a SyRF project. The SyRF environment is designed specifically for the tasks of screening studies for inclusion, assessing study quality, annotating studies and reconciling differences in annotations between reviewers. Registered users can create projects; manage project users; manage bibliographic searches, import references, set up automatic periodic searches; customise annotation questions; categorise and annotate studies; reconcile data and export data for analysis.

The SyRF application is free to the public, so anyone can create a project for themselves. The user who creates the project is usually seen as the project owner and is automatically a part of the project administrator membership group, but this role can be passed to others. Once registered, users can create a public project—which can be viewed by any SyRF user—or a private project, which limits the visible information and requires users to request to join the project. Projects are secured by requiring both a registration request from the user and approval from a project administrator. Project administrators can approve other users and assign them into member groups within a project. In SyRF, a user may be associated with multiple projects and a project may have multiple members reviewing it. This means that multiple users can work on the same project both simultaneously and independently, blinded to the decisions and annotations of others.

### Selection of relevant studies

#### Identification of potentially relevant studies

SyRF supports the importing of systematic searches using several file formats, including XML (EndNote output format) and spreadsheets in tab/comma-delimited value formats (CSV, TSV and TXT). These are easily uploaded by the project administrator and to facilitate review updates there is no limit on how many searches that can be uploaded to the same project. We also support ‘living’ systematic searches, where researchers can create a project with a defined search string, and SyRF will regularly automatically retrieve publications. Users can batch upload PDFs identified in their systematic search for full-text screening and these are automatically matched to records within a project using a file location link specified by the user at the time of uploading. A PDF reupload option is in development, for situations where PDFs held within a project for a certain study are occasionally incorrectly matched, not available at initial upload, or superseded (for instance, when a preprint is after published in full). This is especially beneficial for large-scale collaborative projects where reviewers can highlight and submit PDF corrections for project administrators to review and accept or reject the upload suggestion.

Once you have populated a project with your search results, a project workflow is organised into component tasks called ‘Stages’. Each stage can involve one or more tasks of: screening of publications, metadata annotation of studies using tailor-made questions or extraction of outcome data. Stages can be named and customised for the specific needs of the user and their research question. The order and relation of stages are specified by the project administrator.

#### Selection of relevant studies

To improve the accuracy of the reference selection stage, we recommend that a minimum of two reviewers independently screen each reference for inclusion. The default criteria are for a minimum number of screenings of two and a minimum agreement between two out of three reviewers. This can, however, be adjusted based on the requirements of the project. When an individual begins screening, they are presented with the Title and Abstract, and link to PDF if this is held, of a manuscript drawn at random from those which require screening and for which they have not previously given a screening decision. Inclusion and exclusion criteria are shown in a sidebar. Records remain in the screening pool until final screening disposal (in the usual case, with agreement between two reviewers or, if they disagree, a majority vote of three screeners). All screening decisions are blinded to the decision of other screeners. Further, more detailed classifications with notes (ie, using full study text) can be added to a screening stage by including an annotation field, as described below.

### Metadata annotation and reconciliation

In SyRF, the annotation questions design is very flexible and can be customised for each project. Project administrators can create any question, including metadata annotation questions such as ‘What is the species?’, ‘What is the disease modelled?’, as well as risk of bias questions such as ‘Is the treatment randomly allocated?’. Questions can be nested, so that a question can be followed by a supplementary ‘child’ question, which can either always be asked in the context of a parent annotation or conditionally asked depending on the response to the ‘parent’ question. For example, the options of the question ‘What is the strain?’ can depend on the answer to the question ‘What is the species?’. The flexibility of the annotation questions design allows SyRF to support various types of systematic reviews, including those working towards the Preferred Reporting Items for Systematic Reviews andMeta-Analyses checklist.^2^

Answers can be prespecified using drop-down lists, radio buttons, free-form text fields, and tick boxes, and allow for different types of input. Other choices allow for selecting whether multiple responses to the same question are permitted and whether answers are optional or required. The latter is especially useful for validation of user input, such that if a required answer is not provided, the annotation session is considered incomplete and cannot be submitted until this omission is corrected.

Each stage can include (or consist entirely of) annotation, allowing for more information or characteristics to be recorded from each study. We recommend that at least two researchers annotate each characteristic for each study to minimise errors and reduce subjectivity. In the dual-annotation process, the reconciliation step requires a third reviewer to review the previous reviewers’ answers and provide a final decision or a decision to be made through discussion between the previous reviewers.

### Quantitative outcome data extraction and reconciliation

Many different types and structures of data are presented in preclinical research depending on the type of research being described. Publications often present multiple experiments with different outcome measures, assessed at different time points and often with multiple cohorts of animals. We designed and refined the data extraction process in SyRF to be flexible to suit these different experimental designs.

Users are first asked to provide details of the animal models used, the interventions given, and how these are combined to define different experimental groups of animals. A typical experiment may investigate a disease model by comparing a cohort of disease model animals to a cohort of sham animals. Others may test an intervention by comparing a cohort of disease model animals receiving the intervention with another cohort of disease model animals that did not receive the intervention. Users indicate the outcomes which have been measured and the time(s) at which they have been measured.

For data extraction, SyRF users record the units in which the outcome is reported, the central tendency (usually the mean or the median) for that outcome in that cohort at that time point, along with a measure of dispersion (such as SD or SE of the mean), and the number of experimental subjects contributing to that outcome. To facilitate extraction of data where multiple outcomes are measured at multiple times, we have developed a matrix format to collect aggregated measures for each outcome including the time of outcome assessment, outcome measure value and variance. Preclinical data may be presented in plots, tables or text, and we are concerned that data extraction from plots may be a source of error. We have therefore developed and will shortly release a graph data extraction interface, as described below.

As with other SyRF functions, we recommend that at least two investigators extract data from each publication independently. Reconciliation of outcome data are usually completed by automatically comparing the numeric outcomes and a third reviewer reviewing those outcomes with differences exceeding a certain threshold. In practice, reconciliation of responses here is more complex, requiring first matching of the cohorts for which the outcome has been measured, and second a decision about how closely a continuous variable has to match for the average of two responses to be sufficient, and where further data extraction is required. At present, this requires data to be exported from SyRF, but future releases of SyRF will include embedded reconciliation of metadata and quantitative outcome data.

Fine-grained permission control of project stages by project administrators will be released shortly. This will allow specific tasks for a set of studies within a stage to be assigned to specific membership groups. This may be especially relevant in large crowdsourced projects.

## Supporting implementation of automation in systematic reviews

One of the main motivations behind the development of SyRF was to facilitate the introduction and integration of the use of automated tools as part of the systematic review process. We have developed a series of automation tools, endpoints and methodologies to support large-scale reviews, continuing (‘living’) reviews and risk of bias (study quality) assessment.

The SyRF application architecture is designed to be adaptable and allow the implementation of tools to support living systematic reviews, and some of these tools are already enabled. ‘Living’ reviews are reviews that update continually so that once defined and initiated they can automatically collect, filter, extract, analyse, summarise and disseminate new findings as new evidence becomes available in the literature. They provide a continuously updated summary of evidence for decision making and monitoring the progress of research.

These require automation of at least six components: (1) data retrieval from online archives and depositories, (2) reference selection, (3) metadata annotation, (4) quantitative outcome data extraction, (5) analysis, and (6) visualisation. We have been testing and using development versions of different methods and technologies in the context of our ongoing reviews, with the intention that when performance meets a predetermined threshold these tools can be implemented in the public release of SyRF, with users able to choose to apply these in their projects. SyRF can be used as a platform for tool evaluation and validation.

### Automated data retrieval: living search

Automated data retrieval has been enabled for PubMed in the current version of the app, and we are planning to extend this feature to other electronic reference databases starting with BioRxiv in the next release. This function allows SyRF project managers automatically to retrieve, with configurable frequency, new records matching a predefined search string.

A deduplication function compares PubMed identification numbers every time a new search runs, before adding additional studies to a project. The function is Azure WebJobs programmed with.NET framework and hosted on Azure. This feature will be extended to allow searching across multiple reference databases using an in-house deduplication tool (https://camarades.shinyapps.io/RDedup/, code repository can be found at https://github.com/camaradesuk/ASySD). This tool is built using the RecordLinkage R package,^3^ and existing preclinical systematic review data.

### Reference selection using machine learning

We have created an on-request facility to use the machine learning algorithms for citation screening developed at the EPPI-Centre, UCL^4^ to assist screening on SyRF for studies of relevance specific to individual projects. In our experience, this becomes most useful in projects where more than 5000 citations require screening. Algorithm performance is enhanced by the addition of a human error analysis stage.^5^

### PDF retrieval

We are comparing the efficiency of different approaches (using Zotero at www.zotero.org and CrossRef at www.crossref.org, respectively) to automating PDF retrieval and upload. We will then incorporate the most effective approach as a microservice, recognising the challenges of ensuring that for non-open access publications, such a system takes account of the journal subscriptions held by the project team. Such microservices are independent from SyRF but can be called from the SyRF platform without any expert knowledge.

### Metadata annotation using text mining

We have developed and integrated a risk of bias auto-detector in SyRF that can identify reporting of blinding, randomisation and power calculations in publications describing preclinical studies where we have a full-text PDF.^6^ We have also created text mining dictionaries for the categorisation of publications by disease models reported, therapeutic interventions and outcome measures reported. At present, their deployment is limited to in-house projects, but they will be part of a future SyRF release.

### Data extraction: Graph2Data

Data needed for meta-analyses are often presented graphically within publications. There has been a need for more convenient tools to extract these data. We have previously shown that customised graphical data extraction tools may increase the accuracy and speed at which reviewers can obtain data from graphical representations within manuscripts.^7^ Following the study, we have developed Graph2Data, an open-source web component that can be used to assist users in extracting data from graphs, contained in images. The component (https://eppi-centre.github.io/Graph2Data2/) is designed as a self-contained unit that can be integrated within any web application and will be deployed in a future public SyRF release.

### Data analysis and visualisation

While SyRF does not at present have an integrated analysis feature so to improve analysis efficiency and quality, we have developed a stand-alone web application using a Shiny package in R programming language (https://camarades.shinyapps.io/meta-analysis-app-syrf/), which takes the standard output from SyRF outcome extraction data as its input (code repository available at https://github.com/qianyingw/meta-analysis-app-syrf). The analysis web application includes functions such as meta-analysis, subgroup analysis, metaregression, trim and fill analysis, Egger’s regression and other statistical tests. Similarly, we are developing web interfaces to allow visualisation of information collected in living systematic reviews, including preclinical models of depression (https://camarades.shinyapps.io/Preclinical-Models-of-Depression/), Alzheimer’s disease (https://camarades.shinyapps.io/LivingEvidence_AD/) and chemotherapy-induced peripheral neuropathy (https://khair.shinyapps.io/CIPN/), which will be an important component of future Systematic Online Living Evidence Summaries.

## Usage

**Figure 3:**
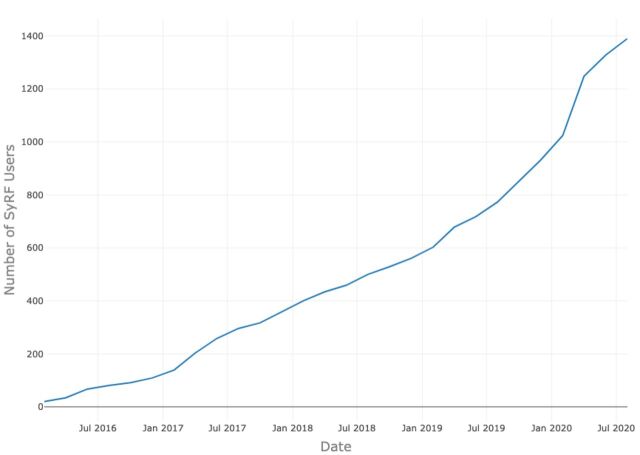
Number of users registered on Systematic Review Facility (SyRF) from launch to date

**Figure 4:**
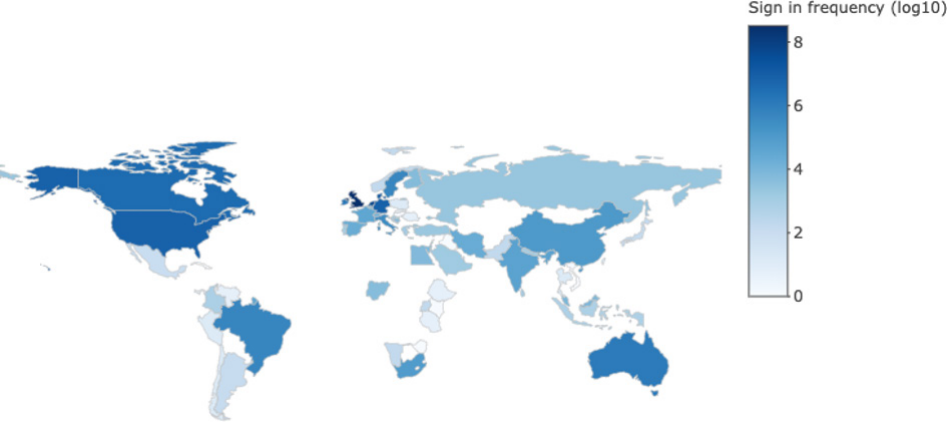
Geographical spread of users accessing Systematic Review Facility.

As of 5 June 2020, SyRF has 1251 registered researchers (figure 3) accessing SyRF from 65 countries across 5 continents (figure 4).

**Figure 5:**
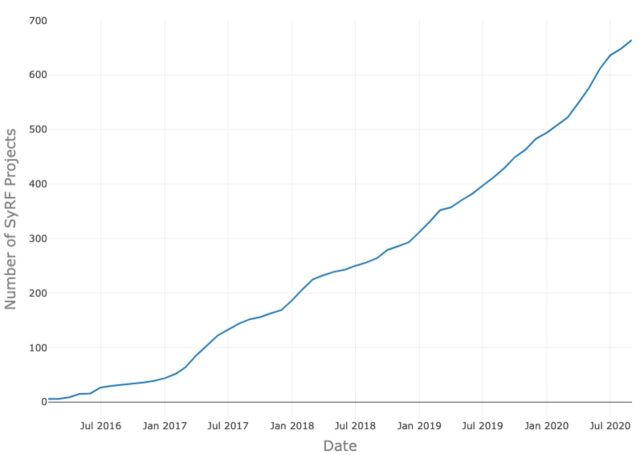
Number of projects created on Systematic Review Facility (SyRF) from launch to date.

Together they are engaged in 588 projects (figure 5), with almost 2 million citations uploaded. While the majority of projects involve only small teams, the crowdsourcing capacity is important, with 24% (141) involving more than two and 6% (34 projects) involving more than 8 investigators.

Challenges involved with having a large group of people with different research backgrounds contribute to a single project include participant retention.^8^ One further benefit of SyRF over traditional methods of systematic review management is that there is a clear audit trail for contributions and decisions made by different reviewers on a project. This allows individual users to see their progress on a project when reviewing and the project administrator has an overview of progress at all times. For crowdsourced projects especially, we have found that sharing progress reports with screeners, for example, can be a helpful way of encouraging participation and keeping a crowd engaged. Moreover, SyRF enables users conducting crowdsourced projects to create training projects that can help familiarise volunteers with studies and questions and thus offer a quality assurance step in managing some of these larger projects.

### Use cases

SyRF, with its robust features and high flexibility, has facilitated various systematic review projects in different research areas including stroke, depression, Alzheimer’s disease and neuropathic pain. One early example of a project utilising our tool is a systematic review examining the efficacy of interleukin-1 receptor antagonist in animal models of stroke, published in 2016, where SyRF was used as the collaborative reference selection tool.^9^ Another project looking at animal models of adverse cardiac remodelling after transverse aortic constriction,^10^ conducted title/abstract screening, full text reference selection, metadata and outcome data extraction in SyRF. And finally, a more recent example of a crowdsourced project has been looking at the link between cannabinoids and pain, where SyRF was used for reference selection, metadata and outcome data extraction, as well as to develop and implement training projects for the crowd. One hundred reviewers were able to contribute to the project through SyRF.^11^

A number of other projects have used SyRF to conduct their systematic review, including the Innovative Medicine Initiatives EQIPD project reviewing guidelines that guide design, conduct and analysis of preclinical biomedical studies,^12^ more topic-specific systematic reviews,^13 14^ and another recent large crowdsourced project summarising primary COVID-19 research,^15^ project progress, results and data available at https://camarades.shinyapps.io/COVID-19-SOLES/.

### Comparison with other systematic review tools

We recognise the existence of a number of widely used and successful systematic review tools available in the public domain today,^16 17^ although SyRF is the only tool specifically developed for systematic review of animal studies. We have included an updated, non-systematic comparison between SyRF and some of these other tools (see table 1), summarising coverage of the systematic review workflow and some of the features we consider to be important in supporting the preclinical systematic review workflow. While some of our features remain under active development, we currently provide a wide coverage of the systematic review workflow as a free service. The exact choice of which software is most appropriate will depend on the user and the requirements of the project itself.

**Table 1:** Comparison between different systematic review software

		SyRF	EPPI-Reviewer 4	SWIFT-Active Screener	Distiller SR	Abstrackr	Rayyan	Covidence	Colandr
Original scientific purpose*		Primarily designed for preclinical reviews	Primarily designed for health-related reviews	Primarily designed for environmental health reviews	Primarily designed for clinical reviews	Primarily designed for clinical reviews	Primarily designed for clinical reviews	Primarily designed for clinical reviews	Primarily designed for conservation research reviews
Phases Supported	Search/upload studies	Yes	Yes	Yes	Yes	Yes	Yes	Yes	Yes
	Deduplicate	Yes, external†	Yes	Yes	Yes	Yes	Yes	Yes	Yes
	Pdf retrieval/upload	Yes	Yes	No	Yes	No	Yes	Yes	Yes
	Screening	Yes	Yes	Yes	Yes	Yes	Yes	Yes	Yes
	Annotation	Yes	Yes	Yes	Yes	Yes	Yes	Yes	Yes
	Outcome data extraction	Yes	Yes	No	Yes	No	No	Yes	No
	Analysis	Yes, external‡	Yes	No	No	No	No	No	No
	Visualisation of analyses	Yes, external‡	Yes	No	No	No	No	No	No
Features	Free to use	Yes	No	No	No	Yes	Yes	Free trial available	Yes
	Technical user support	Yes	Yes	Yes	Yes	Yes	Yes	Yes	Yes
	Methodological user support	Yes, online resources and dedicated helpdesk	No	No	Yes, user community, team to help with training and adoption of best practices and online resources	No	No	Yes	No
	Multiple user support	Yes	Yes	Yes	Yes	Yes	Yes	Yes	Yes
	Reference importing	Yes	Yes	Yes	Yes	Yes	Yes	Yes	Yes
	Reference allocation	Random by default, but can be user adjusted	User defined sorting or machine learning prioritised order	Order reprioritised by likelihood of inclusion based on previous decisions	Order random or possible to reprioritise by likelihood of inclusion based on previous decisions, or user defined	Random or option to reorder by likelihood of inclusion based on previous decisions	User defined order through selection from a list	Can filter with screening or sort using Author, Title, Recency or Relevance	Order reprioritised by likelihood of inclusion based on previous decisions
	Citation screening	Yes	Yes	Yes	Yes	Yes	Yes	Yes	Yes
	Storage and attachment of PDF files	Yes, batch upload possible	Yes, but individual upload required to link to reference list	Yes, but individual upload required unless PDFs directly from EndNote library	Yes, batch upload possible	No	Yes, but individual upload required	Yes, batch upload possible	Yes, but individual upload required
	Publication annotation	Yes	Yes	Yes	Yes	Basic tagging and commenting only	Basic tagging only. With suggestive labelling	Yes	Yes, with suggestive labelling
	Risk of bias assessment	Yes	Yes	Yes	Yes	No	No	Yes	Yes
	Quantitative outcome data extraction	Yes	Yes	No	Yes	No	No	Yes	No
	Annotation fields customisable	Yes	Yes	Yes	Yes	No	No	Yes	Yes
	Discrepancy resolving of screening results	Yes, automated according to user needs	Yes, manual	Yes, manual	Yes	Yes, manual	Yes, manual	Yes, manual	Yes, manual
	Discrepancy resolving of annotation results	Under active development	Yes, manual	No	Yes, manual	No	No	Yes, manual	No
	Exporting results	Yes, Various data structures of download in.csv	Yes	Yes	Yes	Yes	Yes	Yes	Yes
	Multiple user roles	Yes	Yes	Yes	Yes	Yes	Yes	Yes	Yes
	Assign reviewers to study stages	Under active development	Yes	No	Yes	No	No	No	No
	Project management and auditing for administrator	Yes	Yes	Yes	Yes	Yes	Yes	Yes	Yes
	Project and reviewer progress tracking for all users	Yes	Yes	Yes	Yes	Yes	Yes	Yes	Yes
	Flow diagram creation	No	Yes	No	Yes	No	No	Yes	No
	Compatibility with and incorporation of machine learning/automation tools	Yes	Yes	Yes	Yes	Yes	Yes	Yes	Yes
	Keyword highlighting	No	Yes	Yes	Yes	Yes	Yes	Yes	Yes

Data collated from previous publications on systematic review tools^16 17^ and public documentation of each software.

*Most tools will be configurable to multiple types of reviews.

†Available at https://camarades.shinyapps.io/RDedup/.

‡Available at https://camarades.shinyapps.io/meta-analysis-app-syrf/.

SyRF, Systematic Review Facility.

## Conclusion

Preclinical systematic reviews are invaluable but can be incredibly resource-intensive projects, which researchers typically have to proceed with limited domain-specific guidance and without purpose-built project management systems. SyRF was designed to answer the challenges associated with performing these large-scale reviews. As researchers at the forefront of this field, we at CAMARADES perform reviews, guide other researchers performing SRs and actively develop, test and incorporate new technology into our work. Our solution is now being used by over 1200 researchers worldwide. We aim to provide a hub for researchers wanting to perform new, or collaborate on ongoing, systematic reviews of preclinical studies; and those wanting to develop and test new automated tools to incorporate as part of the review process. While we describe SyRF here in the context of preclinical studies, there is no restriction on the type of reviews for which SyRF can be used. We recognise that each project is different and requires slightly different configurations in terms of what data are extracted and analysed. For these reasons, SyRF has been designed to be flexible and the user can define everything from who can review on a project to what questions are asked at each stage of the review process. We will continue to guide researchers in best practice informed by our own and others’ work and experience so that reviews produced are robust and reproducible. We are continually striving to improve our application to better serve the systematic review community and make faster evidence-based decisions in research possible. We strongly support an open-source software model and community collaboration. We are working towards a fully open-source application.

## Data Availability

No data are available. There are no data in this work.
